# Complete Genome Sequence of Corynebacterium ulcerans Strain TSU-28, Harboring Two Diphtheria Toxin Genes, Isolated from a Patient with Diphtheria-Like Symptoms

**DOI:** 10.1128/mra.00072-23

**Published:** 2023-05-03

**Authors:** Jun Kawase, Tsuyoshi Sekizuka, Tomotake Sakai, Naoki Fujisawa, Masaaki Iwaki, Miyuki Kimura, Makoto Kuroda

**Affiliations:** a Division of Bacteriology, Shimane Prefectural Institute of Public Health and Environmental Science, Matsue, Shimane, Japan; b Laboratory of Bacterial Genomics, Pathogen Genomics Center, National Institute of Infectious Diseases, Tokyo, Japan; c Shimane Prefectural Hamada Public Health Center, Hamada, Shimane, Japan; d Department of Bacteriology II, National Institute of Infectious Diseases, Tokyo, Japan; e Management Department of Biosafety, Laboratory Animal, and Pathogen Bank, National Institute of Infectious Diseases, Tokyo, Japan; University of Rochester School of Medicine and Dentistry

## Abstract

Diphtheria toxin-producing Corynebacterium ulcerans is an emerging zoonotic pathogen that causes severe disease in humans. Here, we report the complete genome sequence of C. ulcerans strain TSU-28, harboring two diphtheria toxin genes, which was isolated from the throat of a patient with diphtheria-like symptoms in 2019 in Japan.

## ANNOUNCEMENT

Diphtheria toxin (DT)-producing Corynebacterium ulcerans causes diphtheria in humans (1). DT is encoded by a DT gene (*tox*)-positive prophage in the chromosome (2, 3); no C. ulcerans strain harboring two DT genes has been reported to date. Here, we report the complete genome sequence of C. ulcerans strain TSU-28, harboring two DT genes, which was isolated from a diphtheria patient in 2019 in Japan.

TSU-28, which was received from a hospital in Shimane prefecture in 2019, was streaked on 5% sheep blood agar (SBA) (Kyokuto, Japan) and incubated overnight at 37°C. A single colony was streaked again on SBA to yield a pure culture before being stocked at −80°C in 10% skim milk. TSU-28 was identified as toxigenic C. ulcerans by real-time PCR and Vero cell assay (4, 5) using the pure culture. The frozen stock was plated on SBA, and a single colony was inoculated into brain heart infusion (BHI) broth (Difco) following overnight growth at 37°C. DNA extracted from overnight cultures in BHI broth at 37°C using Genomic-tip 100/G columns (Qiagen, Germany) was used for both Illumina and Nanopore sequencing. Genomic libraries were prepared using a DNA preparation kit (Illumina, San Diego, CA) with an insert size of 300 to 350 bp and the ligation sequencing kit SQK-LSK109 (Oxford Nanopore Technologies, Oxford, UK) without size selection or shearing. Illumina sequencing and Nanopore sequencing were performed using the Illumina iSeq 100 system with a 2 × 150-bp paired-end protocol and the Oxford Nanopore Technologies MinION MK1C instrument with the flow cell R9.4.1 (FLO-MIN106D), respectively. Default parameters were used for all software except where otherwise noted. The Illumina raw reads were adapter trimmed with Local Run Manager software v2.4.0 (Illumina). Base calling and adapter trimming for Nanopore raw reads were performed using Guppy v5.1.12/MinKNOW v21.11.6 (Oxford Nanopore Technologies). Quality filtering using the adapter-trimmed Nanopore and Illumina reads was performed (quality score limit, 5%) using the Trim Reads tool in CLC Genomics Workbench v21.0.5 (CLC21.0.5) (Qiagen). The filtered Nanopore reads were assembled using CLC21.0.5 (*De Novo* Assemble Long Reads tool), followed by polishing using CLC21.0.5 (Polish with Reads tool) with the filtered Illumina reads. The circular contig, which was confirmed by CLC21.0.5 (Assembly Graph tool), was then error corrected using Pilon v1.18 with the filtered Illumina reads (6). The average coverage and GC content for the final contig were calculated with CLC21.0.5 (Map Reads to Contigs tool). DFAST v1.2.3 (7), with the DFAST default database and the Virulence Factor Database (VFDB) (8), was used for gene annotation and the genome rotation to start with *dnaA*. Sequence characteristics are listed in Table 1.

**TABLE 1:** Sequencing data and genomic characteristics of Corynebacterium ulcerans TSU-28

Characteristic	Finding
Illumina sequencing	
Read length (nucleotides)	2 × 150
No. of read pairs	2,424,642
No. of filtered read pairs	2,390,878
Nanopore sequencing	
No. of reads	284,000
Read *N*_50_ (bp)	10,980
No. of filtered reads	92,331
Avg length of filtered reads (bp)	4,512
Complete genome	
No. of contigs	1
Structure	Circular
Total genome length (bp)	2,522,327
GC content (%)	53.4
Avg coverage (×)	
Illumina reads	138
Nanopore reads	160
Predicted no. of coding DNA sequences	2,276
No. of rRNAs (5S, 16S, 23S)	4, 4, 4
No. of tRNAs	54
No. of DT genes	2
BLASTp similarity between the 2 DT genes (%)	99.11
GenBank accession no.	AP027140

**FIG 1:**
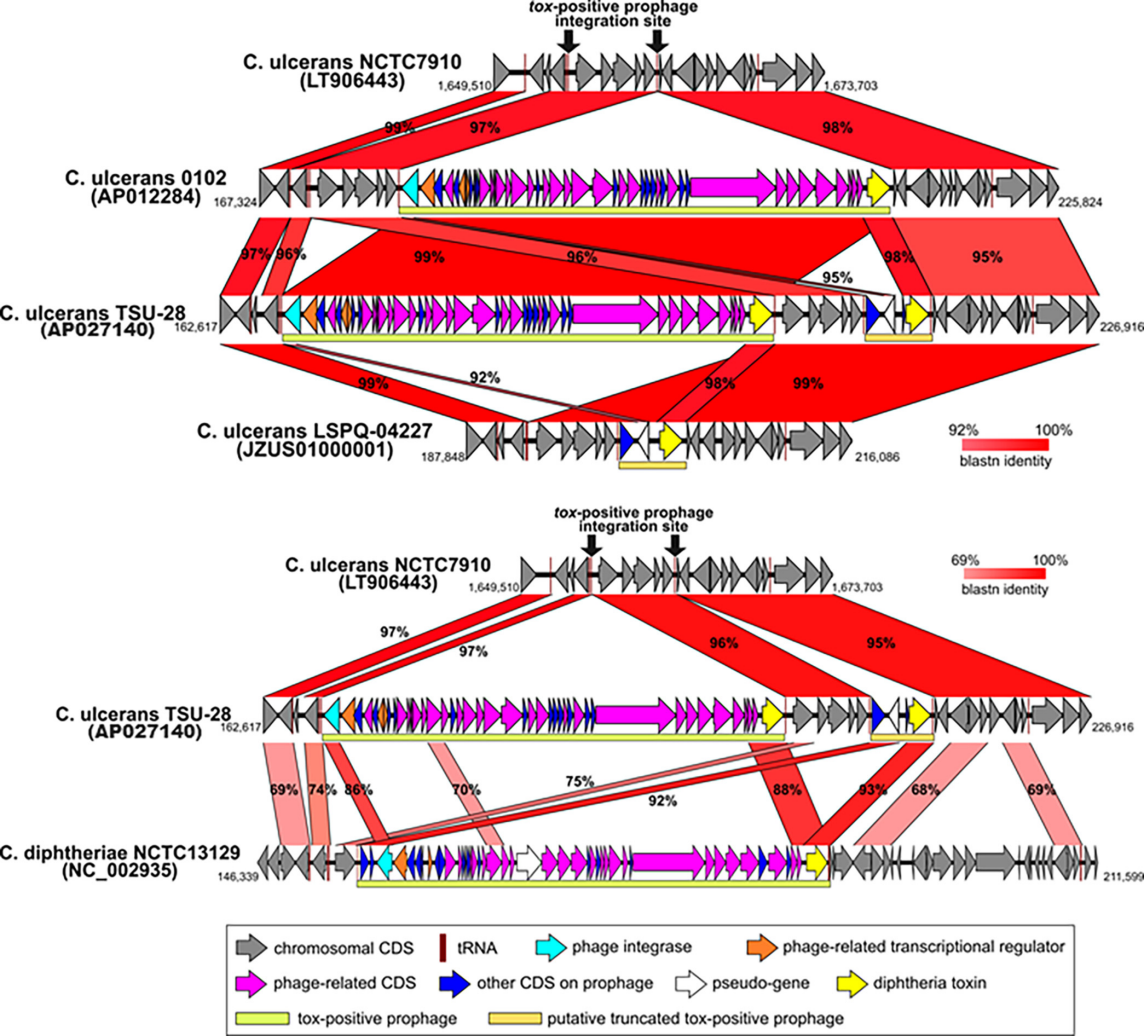
Comparison of *tox*-positive prophages and their adjacent regions built with EasyFig using the BLASTn search tool. The *tox*-positive prophage and adjacent regions of C. ulcerans 0102, TSU-28, and LSPQ-04227 and C. diphtheriae NCTC 13129 are shown. The corresponding region of C. ulcerans NCTC 7910 is also shown. Boxes indicate individual coding regions, with colors assigned to their functions. The numbers in parentheses indicate GenBank accession numbers. CDS, coding sequence.

BLASTn searching showed that the TSU-28 *tox*-positive prophage is identical to the *tox*-positive prophage of 0102 (9) (Fig. 1). Furthermore, the putative truncated *tox*-positive prophage downstream of the TSU-28 prophage conserved the structure of LSPQ-04227 and is highly homologous with the integrase and its upstream sequence in the NCTC 13129 prophage.

The Ethics Committee of Shimane Prefectural Institute of Public Health and Environmental Science approved this study and waived the requirement for informed consent due to the retrospective nature of the study, with anonymization of the patient’s data (approval number 32-PHESI-ET-202203).

### Data availability.

The sequencing data have been deposited in the DDBJ/EMBL/GenBank database under accession number AP027140 for the chromosome. The Illumina paired-end fastq files and Nanopore base-called fastq files are available in the Sequence Read Archive (SRA) under accession numbers DRR426628 and DRR456193, respectively.
